# Transcriptome Profiling of N7-Methylguanosine Modification of Messenger RNA in Drug-Resistant Acute Myeloid Leukemia

**DOI:** 10.3389/fonc.2022.926296

**Published:** 2022-07-05

**Authors:** Bing Zhang, Dong Li, Ran Wang

**Affiliations:** ^1^ Department of Pediatrics, Qilu Hospital of Shandong University, Shandong, China; ^2^ Department of Hematology, Qilu Hospital of Shandong University, Shandong, China

**Keywords:** N7-methylguanosine(m7G), messenger RNA (mRNA), drug-resistant, acute myeloid leukemia (AML), bioinformatics analysis

## Abstract

Acute myeloid leukemia (AML) is an aggressive hematological tumor caused by the malignant transformation of myeloid progenitor cells. Although intensive chemotherapy leads to an initial therapeutic response, relapse due to drug resistance remains a significant challenge. In recent years, accumulating evidence has suggested that post-transcriptional methylation modifications are strongly associated with tumorigenesis. However, the mRNA profile of m7G modification in AML and its role in drug-resistant AML are unknown. In this study, we used MeRIP-seq technology to establish the first transcriptome-wide m7G methylome profile for AML and drug-resistant AML cells, and differences in m7G between the two groups were analyzed. In addition, bioinformatics analysis was conducted to explore the function of m7G-specific methylated transcripts. We found significant differences in m7G mRNA modification between AML and drug-resistant AML cells. Furthermore, bioinformatics analysis revealed that differential m7G-modified mRNAs were associated with a wide range of cellular functions. Importantly, down-methylated m7G modification was significantly enriched in ABC transporter-related mRNAs, which are widely recognized to play a key role in multidrug resistance. Our results provide new insights into a novel function of m7G methylation in drug resistance progression of AML.

## Introduction

Acute myeloid leukemia (AML) is a heterogeneous malignant clonal disease caused by the proliferation of hematopoietic stem or progenitor cells by gene mutation. Approximately 50% of young and middle-aged patients and over 80% of elderly patients with AML die each year due to primary drug resistance, relapse after remission, and treatment-related complications (1). Although a variety of new drugs are currently undergoing clinical trials, AML therapies have not yet broken through the classic treatment of anthracyclines combined with cytarabine. Although allogeneic hematopoietic stem cell transplantation is the most effective method for AML treatment, the limitation of suitable populations restricts its application (2). Therefore, there is an urgent need for drug development to improve the overall survival rate and quality of life of AML patients. In the past decade, researchers have identified many recurrent gene mutations involved in the coding of epigenetic regulatory proteins through gene sequencing, suggesting that the occurrence of leukemia is closely related to abnormal epigenetic modifications and that abnormal RNA methylation is an important mechanism (3, 4).

RNA methylation regulates gene expression at the post-transcriptional level and is considered another layer of epigenetic regulation similar to DNA methylation and histone modification. At present, over 150 RNA methylation modifications have been identified as post-transcriptional regulatory markers in eukaryotes, and these modifications can regulate biological metabolic processes such as RNA alternative splicing, nuclear export, stabilization, and translation (5, 6). Methylation modifications of RNA mainly include N6-methyladenosine(m6A), 5-methylcytosine C5-methylcytidine(m5C), N1-methyladenosine(m1A), and 7-methylguanosine (m7G). Among these, the m7G modification is one of the most common methylation modifications in post-transcriptional regulation and is widely distributed in the 5’cap region of tRNA, rRNA, and mRNA in eukaryotes. This modification occurs during the initial stages of transcription and before other RNA-processing events (7).

Recent studies have confirmed that m7G modification is involved in the regulation of multiple processes such as mRNA transcription, splicing, nuclear export, and translation (8, 9). However, while m5C and m6A methylation modifications have been widely studied in many diseases (10, 11), few studies have been conducted on m7G methylation. In recent years, high-throughput sequencing methods for m7G modifications within the transcriptome have gradually advanced (12, 13). In 2019, Zhang et al. developed m7G-MeRIP sequencing (MeRIP-seq) for transcriptional profiling of m7G methylation in human cells (14).

To further investigate the role of m7G in drug resistance in AML cells, we used MeRIP-seq technology to establish the first known transcriptome-wide m7G methylome profile for the AML cell line HL60 and AML drug-resistant cell line. The mRNA levels in HL60 and HL60/MX2 cells were characterized by m7G-specific analysis and in-depth bioinformatics analysis. Additionally, RNA-seq was used to detect differentially expressed genes, and a combined analysis of differentially methylated and expressed genes was performed.

In this study, our results showed that there were significant differences in the genes modified by m7G methylation in AML-resistant cells, and the number of methylated genes and peaks in drug-resistant cells was greater than those in non-resistant cells. In AML drug-resistant cells, a large number of tumor-related gene m7G methylation changes can result in altered mRNA expression. Bioinformatics analysis revealed that the methylation of the two groups was different, which may lead to variable changes in cell function. Our study suggests a possible association between AML and m7G methylation in mRNA and predicts the functional changes that may arise from the difference in m7G methylation, which could be a breakthrough for improving AML treatment.

## Materials and Methods

### Cell Lines and Cell Culture

Human promyelotic HL60 and HL60/MX2 cells were obtained from the American Type Culture Collection. The cells were grown in RPMI-1640 (Gibco, USA) supplemented with 10% heat-inactivated fetal calf serum (Gibco, USA) and 1% (v/v) antibiotics (penicillin-streptomycin) (Gibco, USA) in a humidified 5% CO_2_ atmosphere at 37°C (15).

### RNA Extraction and Fragmentation

Cells in the logarithmic growth phase were collected, total RNA was extracted using TRIzol reagent (Invitrogen Corporation, CA, USA) following the manufacturer’s instructions, and RNA concentration was measured using a NanoDrop ND-100 (Thermo Fisher Scientific, Waltham, MA, USA). The purity of the RNA was evaluated based on its OD260/OD280 ratio. If the OD260/OD280 value range was 1.8 ~ 2.1, the RNA purity was qualified. Denaturing agarose gel electrophoresis was used to measure the RNA integrity and gDNA contamination.

### Merip Library Construction and Sequencing

m7G-IP-Seq service was provided by CloudSeq Inc. (Shanghai, China). mRNA was isolated from total RNA with oligo(dT) magnetic beads (ThermoFisher) and subjected to immunoprecipitation using the GenSeqTM m7G-IP Kit (GenSeq Inc., China) according to the manufacturer’s instructions. Briefly, RNA was decapped with tobacco decapping enzyme and randomly fragmented to ~200 nt using RNA fragmentation reagents. Protein A/G beads were coupled to the m7G antibody by rotation at room temperature for 1 h. RNA fragments were incubated with bead-linked antibodies and rotated at 4°C for 4 h. The RNA/antibody complexes were then digested with proteinase K, and the eluted RNA was purified by phenol:chloroform extraction. RNA libraries for IP and input samples were constructed using the NEBNext Ultra II Directional RNA Library Prep Kit (New England Biolabs, Inc., USA) according to the manufacturer’s instructions. Libraries were qualified using an Agilent 2100 Bioanalyzer and sequenced on a NovaSeq platform (Illumina).

### Sequencing Data Analysis of m7G Modification Peaks

Paired-end reads were harvested from the Illumina NovaSeq 6000 sequencer and quality was controlled by Q30. Cutadapt software (v1.9.3) was used for 3’ adaptor trimming, removing low-quality reads, and obtaining high-quality clean reads (16). First, clean reads of the input libraries were aligned to the reference genome (UCSC HG19) using STAR software (17). Then, mRNAs were identified by DCC software using STAR alignment results (18). Next, clean reads from all libraries were aligned to the reference genome using Hisat2 software (v2.0.4) (19). Methylated sites on mRNAs (peaks) were identified using MACS software (20). Differentially methylated sites were identified using diffReps (21). The peaks identified by both software overlapping with the exons of the protein-coding genes were identified and chosen by homemade scripts.

Dreme software was used to detect the sequence of the methylation peaks to identify meaningful motifs. The E value of the motif is the enrichment P value multiplied by the number of candidate motifs tested; the enrichment P value uses Fisher’s exact test method to enrich the motifs in the positive sequence. The E value of the motif is negatively correlated with its credibility; that is, the lower the E value, the higher the credibility. GO analysis and KEGG pathway enrichment analysis were performed to determine the genes of differentially methylated mRNAs.

### Transcriptome Sequencing Analysis and Statistical Analysis

The high-quality reads were aligned to the human reference genome (UCSC hg19) using hisat2 software (v2.0.4). Guided by the Ensembl gtf gene annotation file, cuffdiff software (v2.2.1, part of cufflinks) was used to obtain the FPKM as the expression profiles of mRNA, and fold change and p-value were calculated based on FPKM to screen differentially expressed mRNAs. Fold change ≥ 2, p-value ≤ 0.05, and FPKM value ≥ 0.5 in at least one sample were used as the criteria for screening differential mRNA.

Statistical analysis was performed using SPSS 25.0 and GraphPad Prism 8.0. Student’s two-tailed t-test was used to compare significant differences between the two groups. Differences were defined as statistically significant at p < 0.05.

## Results

### General Features of m7G Methylation in HL60 and HL60/MX2 Cells

**Figure 1:**
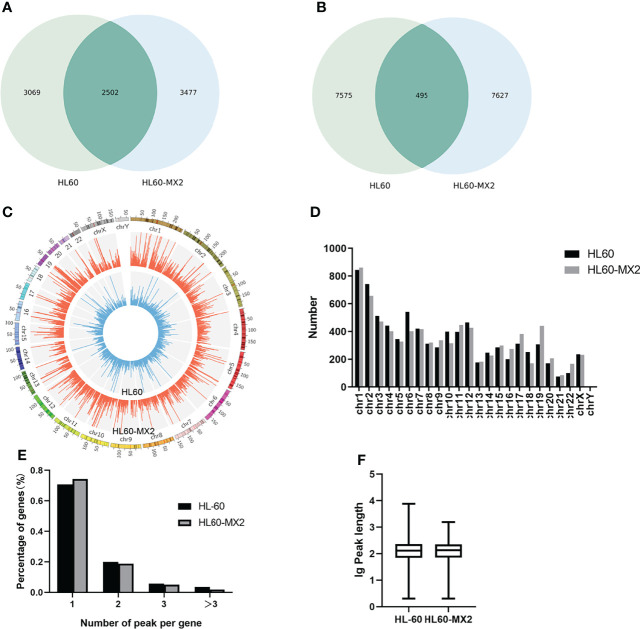
Overview of m7G methylation in HL60 and HL60/MX2 cells. **(A)** Venn diagram of m7G genes in HL60 and HL60/MX2 cells. **(B)** Venn diagram of m7G peaks in HL60 and HL60/MX2 cells. **(C)** Visualization of m7G at the chromosome level in HL60 and HL60/MX2 cells. **(D)** The number of m7G peaks in HL60 and HL60/MX2 cells on each chromosome. **(E)** The number of m7G peaks on each mRNA. **(F)** The length of the m7G methylation peaks in HL60 and HL60/MX2 cells.

RNA sequencing and transcriptome-wide m7G methylation sequencing assays were performed in HL60 and HL60/MX2 cells. In the HL-60 group, we found 8070 clean methylation peaks representing transcripts of 5571 genes. A total of 8122 peaks were identified in the HL60/MX2 group, corresponding to 5979 gene transcripts. Notably, only 495 identical peaks appeared in both groups. HL60/MX2 group had 7627 unique peaks and 7575 missing peaks compared to HL60, indicating that the m7G methylation modification features were significantly different between HL60 and HL60/MX2 cells. The average number of peaks per gene among the unique methylated genes in HL60 cells was 2.47, compared to 2.19 in HL60/MX2 cells (**Figures 1A, B**).

### Distribution Characteristics of m7G Methylation

Analysis of the distribution of m7G peaks of mRNA on different chromosomes found that there were differences in the number and distribution of m7G peaks on each chromosome. Among these, the number of m7G peaks on chromosomes 1 and 2 was the highest and the number of m7G peaks on chromosome 21 was the lowest. From the comparison of m7G peaks between the two groups, the most obvious differences were identified on chromosomes 6 and 19. In addition, autosome methylation levels were generally higher than those of the sex chromosomes in both groups (**Figures 1C, D**). As shown in **Figure 1E**, when we counted the number of m7G peaks for each mRNA in the two groups, we found that most methylated mRNAs in HL60 and HL60/MX2 cells had only one m7G peak (70.8% and 74.3%, respectively). In the two groups, approximately 20% of the genes recorded two m7G peaks, and less than 10% of the genes contained more than three m7G peaks, which was consistent with previous studies of methylation in other diseases (22, 23). We performed a statistical analysis of the length of the methylation peak, and our results revealed that there was no statistically significant difference between the two groups. The average length of the methylation peak was 114.82 nt (**Figure 1F**).

### Analysis of Differentially-Regulated m7G Methylation Genes

To explore the differential expression of m7G methylated genes in HL60 and HL60/MX2 cells, we performed statistical analysis on the m7G-modified genes of the two groups. A difference of more than two times (P ≤ 0.00001) was considered methylation upregulation or downregulation. Compared with HL60 cells, m7G methylation was upregulated in 427 genes and downregulated in 452 genes in HL60/MX2 cells. The top 15 upregulated and downregulated genes are listed in **Tables 1**, **2**.

**Table 1:** Top 15 up-methylated genes (HL60/MX2 vs HL60).

Chromosome	txStart	txEnd	Gene name	Fold change
chr1	1822258	1822556	GNB1	6345.1
chr9	112020456	112020577	EPB41L4B	2873.8
chr6	78400372	78400400	MEI4	2616
chr15	59949013	59949200	GTF2A2	2597.6
chr1	184774757	184775060	FAM129A	2468.7
chrX	47430841	47431220	ARAF	2220.1
chr12	2706602	2706662	CACNA1C	2137.2
chr20	44175905	44175960	EPPIN	2082
chr19	42569374	42569400	GRIK5	2026.7
chr9	35826061	35826158	FAM221B	1962.3
chr21	45457672	45457808	TRAPPC10	1861
chr6	36790833	36790874	CPNE5	1787.3
chr1	160793970	160794042	LY9	1741.3
chr2	84928401	84928463	DNAH6	1566.3
chr9	38395736	38395920	ALDH1B1	1317.7

**Table 2:** Top 15 down-methylated genes (HL60/MX2 vs HL60).

Chromosome	txStart	txEnd	Gene name	Fold change
chr10	50662525	50662720	ERCC6	3552.4
chr7	2338979	2339072	SNX8	3117.9
chr5	31508727	31508760	DROSHA	2629.2
chr11	14316305	14316408	RRAS2	2422.9
chr17	26925482	26925520	SPAG5	2390.3
chr6	160665501	160666480	SLC22A2	2325.1
chr8	7308085	7308422	SPAG11B	2292.5
chr10	69751955	69752080	HERC4	2042.8
chr14	20482881	20483352	OR4K14	1988.5
chr18	10739221	10740000	PIEZO2	1912.4
chr7	150270681	150271041	GIMAP4	1901.6
chr3	148939441	148939832	CP	1771.2
chr18	53070851	53071226	TCF4	1760.4
chr9	102742001	102742360	ERP44	1738.7
chr1	222757469	222757580	TAF1A	1716.9

### Motif Analysis of m7G Methylation

**Figure 2:**
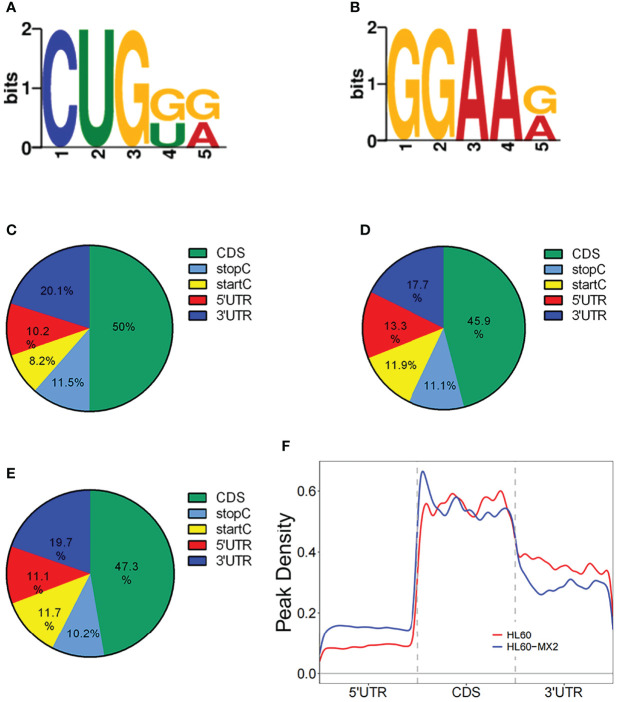
Motif and regional analysis of m7G methylation. **(A)** Motif with minimum E-value of m7G in HL-60 cells. **(B)** Motif with minimum E-value of m7G in HL60/MX2 cells. **(C)** Pie chart of m7G peaks in different regions of mRNA in HL-60 cells. **(D)** Pie chart of m7G peaks in different regions of mRNA in HL60/MX2 cells. **(E)** Pie chart of m7G peaks in different regions of mRNA in differentially modified genes of the two groups. **(F)** Analysis of the peak density modified by m7G methylation.

In this study, DREME software was used to determine the MOTIF sequence. As shown in **Figures 2A, B**, the most conservative MOTIFs were CUGKR (K=G/U, R=G/A) and GGAAR, with E-values of 6.5e-016 and 2.0e-013 in HL-60 cells, respectively. The MOTIFs sequences of the two groups were significantly different.

### Regional Analysis of m7G Methylation

We analyzed the m7G methylation site, and our results showed that the modification was distributed in all mRNA regions. Among these, the CDS region exhibited the most methylation modification, followed by the 3’UTR region, and the StartC and StopC regions demonstrated the least methylation modification (**Figures 2C, D**). The regional distribution of the differentially modified genes in HL60 and HL60-MX2 cells was similar (**Figure 2E**). As shown in **Figure 2F**, we performed a statistical analysis of the peak density modified by m7G methylation. The results showed that the number of m7G peaks in the two groups was similar in the CDS region. Compared to HL60-MX2 cells, HL60 cells showed fewer m7G peaks in the 5’UTR region and more in the 3’UTR region.

### Conjoint Analysis of Differentially Expressed m7G-Methylated Genes and mRNA Expressed Genes

**Figure 3:**
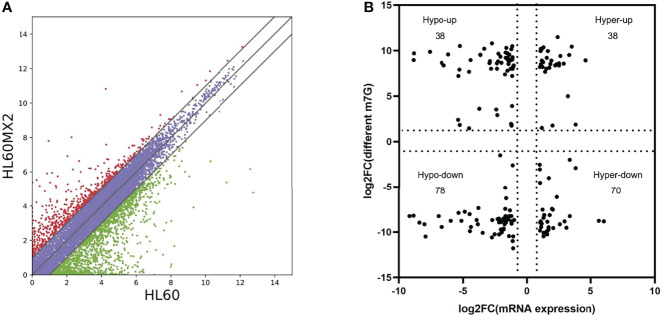
Joint analysis of m7G methylation and transcriptome. **(A)** Scatter plots of the differentially expressed mRNAs between HL60 and HL60/MX2 cells. **(B)** Conjoint analysis of m7G methylation and the transcriptome of mRNA expression.

RNA sequencing analysis revealed that there were statistically significant differences in the expression of 4801 genes in HL60/MX2 cells compared with HL60 cells, among which 1451 genes were upregulated and 3350 genes were downregulated. Scatter plots were used to represent the differentially expressed mRNAs between the two groups (**Figure 3A**). We conducted a conjoint analysis of the degree of m7G methylation and mRNA expression. The results showed that among the 116 genes with low mRNA expression, the degree of m7G methylation was significantly upregulated for 38 genes and significantly downregulated for 78 genes (fold-change > 2, p < 0.001), referred to as ‘hypo-up’ and ‘hypo-down’. Among the 108 genes with high mRNA expression, the m7G methylation degree was significantly upregulated for 38 genes and significantly downregulated for 70 genes, referred to as ‘hyper-up’ and ‘hyper-down’ (**Figure 3B**).

### Bioinformatics Analysis of m7G Methylation Genes

**Figure 4:**
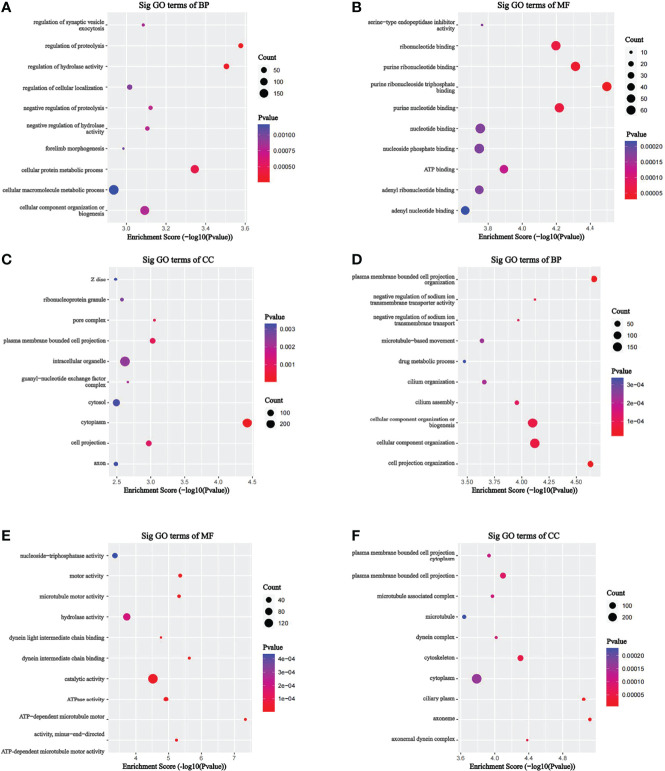
GO analysis on different m7G methylation genes of HL60 and HL60/MX2 cells. **(A-C)** GO enrichment analysis of up-methylated m7G genes in HL60/MX2 cells. **(D-F)** GO enrichment analysis of down-methylated m7G genes in HL60/MX2 cells. GO, gene ontology; BP, biological process; CC, cellular component; MF, molecular function.

To investigate the pathophysiological role of m7G methylation modification in the induction of drug resistance in AML cells, we performed GO enrichment and KEGG pathway analyses on different m7G methylation genes in HL60/MX2 and HL60 cells. As shown in **Figure 4**, in terms of biosynthetic biological processes (BP), the up-methylated m7G genes in HL60/MX2 cells were mainly related to the regulation of proteolysis, regulation of hydrolase activity, and cellular protein metabolic process, while down-methylated m7G genes were mainly related to plasma membrane-bounded cell projection organization, cell projection organization, cellular component organization or biogenesis. In terms of molecular functions (MF), the up-methylated m7G genes were mainly related to purine ribonucleoside triphosphate binding, purine ribonucleotide binding, purine nucleotide binding, and ribonucleotide binding, while down-methylated m7G genes were mainly related to ATP-dependent microtubule motor, dynein intermediate chain binding, and motor activity. For the cellular components, the up-methylated m7G genes were mainly related to cytoplasm, pore complex, and plasma membrane-bounded cell projection, while down-methylated m7G genes were mainly related to axoneme, ciliary plasm, axonemal dynein complex, and cytoskeleton.

**Figure 5:**
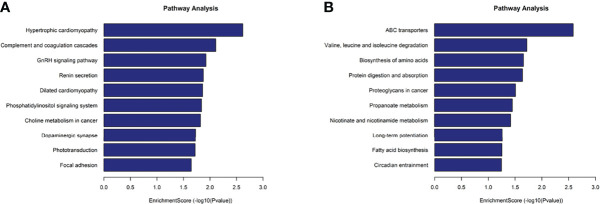
KEGG analysis on different m7G methylation genes of HL60 and HL60/MX2 cells. **(A)** Major enriched KEGG pathway of up-methylated m7G in HL60-MX2 cells. **(B)** Major enriched KEGG pathway of down-methylated m7G in HL60-MX2 cells.

KEGG analysis results revealed that the mRNAs with up-methylated m7G modification in HL60-MX2 cells were primarily involved in hypertrophic cardiomyopathy, complement and coagulation cascades, and the GnRH signaling pathway (**Figure 5A**). The mRNAs with down-methylated m7G modification were significantly enriched in ABC transporters, valine, leucine, and isoleucine degradation, and amino acid biosynthesis (**Figure 5B**). Each graph in **Figures 4**, **5** lists the top ten most dominant genes in each category.

## Discussion

Epigenetic modification of genes is a hot topic in the study of tumor pathogenesis. Recent studies have shown that epigenetic abnormalities, including RNA methylation (24), DNA methylation (25), histone covalent modification (26), and non-coding RNAs, play important roles in the occurrence and development of tumors.

At present, methylation of some genes has been shown to be related to the occurrence and progression of hematological tumors, and the detection of gene methylation is also of great significance to the judgment of prognosis. For example, the presence of DNA hypermethylation of RASSF6 and RASSF10 always indicates a poor prognosis in acute lymphoblastic leukemia (ALL) (27). Methyltransferase-like 3 (METTL3) is a core m6A catalytic enzyme (28). Lin et al. found that, compared with normal hematopoietic stem cells, the mRNA and protein expression levels of METTL3 in leukemia cells of patients with acute myeloid leukemia (AML) were significantly increased. The high expression of METTL13 promoted its target genes, including c-MYC and BCL2, and increased m6A methylation of PTEN-promoted mRNA translation (29).

Recently, m7G methylation modifications have attracted increasing attention. Methyltransferase-like 1 (METTL1) is a writer of m7G methylation (30). Recent studies also reported that METTL1 is involved in tumor development by promoting mRNA processing in an m7G-dependent manner (31). In addition, METTL1 overexpression has been shown to be associated with poor prognosis and downregulation of tumor suppressors in hepatocellular carcinoma (32). In terms of the occurrence, development, and drug resistance of leukemia, there have been many studies on RNA methylation, but they have all focused on m5C and m6A modifications (33–35). Currently, there is a lack of research on m7G methylation modification of mRNA in leukemia.

In this study, we sequenced the m7G methylation peaks of mRNA in AML cells (HL60) and drug-resistant AML cells (HL60-MX2) using MeRIP-seq and analyzed the differences between the two groups. We identified over 10,000 m7G peaks and nearly 10,000 m7G methylated genes and found significant differences in m7G methylation modification status in HL60 and HL60-MX2 cells. The common modified genes were only 27%, the common modification peaks were only ~3%, and the overall methylation level in HL60-MX2 cells was higher than that in HL60 cells. This suggests that m7G methylation may be involved in the regulation of drug resistance in AML cells.

Epigenetic modification is an important area of study in tumor pathogenesis. Recent research has shown that epigenetic abnormalities, including RNA methylation, DNA methylation, histone covalent modification, and miRNA abnormalities, play an important role in the occurrence and development of tumors (26). At present, methylation of some genes has been shown to be related to the occurrence and progression of hematological tumors, and detection of gene methylation is also of great significance to the judgment of prognosis. ARAF, a member of the RAF kinase family, is involved in tumor development by activating mitogen-activated protein kinase (MAPK) in various malignancies including lung, colorectal, pancreatic, and breast cancers (36). Our results showed that ARAF was significantly upregulated in m7G methylation in HL60/MX2 cells. Among genes with downregulated methylation in HL60/MX2, ERCC6 was directly involved in regulating the response to 5-FU chemotherapy in colorectal cancer (37). However, the role of m7G methylation in the above genes in AML pathogenesis and drug resistance remains unclear. In our study, analysis of m7G methylation modification motifs revealed significant differences between drug-resistant AML cells and non-resistant AML cells, suggesting that the differences in methylation modification between the two groups may be related to the category of m7G methylase. However, this still requires further experimental confirmation.

A large number of studies have shown that the distribution of methylation sites in different regions of mRNA is crucial to mRNA stability and translation regulation. Meyer et al. observed that m6A modification sites were enriched near stop codons and in 3’ UTRs, and found that m6A modification enriched in 3’ UTRs could affect the binding of microRNAs, indicating that methylation modification could play a role at the post-transcriptional level (38). A recent study found that a significant increase in HSPA1A mRNA and protein expression is regulated by m6A modification, which may be related to increased m6A methylation in the CDS region of HSPA1A, promoting mRNA stability (39). Our study found that most m7G methylation occurred in the CDS region in both AML and drug-resistant AML cells, and most of the methylation differences between the two groups occurred in the CDS region. Whether m7G methylation affects mRNA stability and expression regulation in AML pathogenesis and drug resistance has not yet been confirmed; therefore, more complex experiments are required for further study.

Multidrug resistance (MDR) has become a major reason for tumor chemotherapy failure (40). MDR refers to the cross-resistance of tumor cells to chemotherapeutic drugs, as well as other chemotherapeutic drugs with different structures and functions (41). The mechanism of MDR is an important topic of basic and clinical tumor research. The expression of drug transport pumps, especially the overexpression of ATP-binding cassette transporters (ABC transporters), is closely related to tumor MDR. Importantly, ABC transporter proteins are considered the major reason for MDR in AML (42). Increasing evidence suggests that altered expression of ABC transporters contributes not only to MDR but also to cancer initiation, progression, and metastasis (43, 44). Previous studies have demonstrated that aberrant DNA methylation of ABC transporters is present in a variety of neoplastic diseases, including MDR leukemia cell lines (45, 46). However, the effect of RNA methylation on ABC transporters remains unclear. Interestingly, our KEGG analysis results showed significant downregulation of m7G methylation in ABC transporter-related genes of HL60-MX2 cells, which may suggest that downregulation of m7G methylation could positively regulate ABC transporter-related genes in AML cells, leading to cell drug resistance. For more than 30 years, researchers have attempted to use MDR inhibitors in cancer chemotherapy without success. However, blocking ABC transporter activity has been shown to be effective *in vitro*, since the toxicity of the inhibitor drug limited its clinical application (47). Our study provides a new perspective for future investigators to study the role of m7G methylation in drug resistance of AML cells to discover new therapeutic targets.

## Data Availability Statement

The datasets presented in this study can be found in online repositories. The names of the repository/repositories and accession number(s) can be found below: https://www.ncbi.nlm.nih.gov/geo/query/acc.cgi?acc=GSE201096.

## Author Contributions

All authors listed have made a substantial, direct, and intellectual contribution to the work and approved it for publication. All authors contributed to the article and approved the submitted version.

## Funding

This work was supported by the National Natural Science Foundation of China (no. 81473484).

## Conflict of Interest

The authors declare that the research was conducted in the absence of any commercial or financial relationships that could be construed as a potential conflict of interest.

## Publisher’s Note

All claims expressed in this article are solely those of the authors and do not necessarily represent those of their affiliated organizations, or those of the publisher, the editors and the reviewers. Any product that may be evaluated in this article, or claim that may be made by its manufacturer, is not guaranteed or endorsed by the publisher.
